# Commentary: ‘Case Report: A Rare Case of Elderly-Onset Adult Onset Still’s Disease in a Patient With Systemic Lupus Erythematous’

**DOI:** 10.3389/fimmu.2022.876477

**Published:** 2022-04-07

**Authors:** Marion Delplanque, Arsène Mekinian, Sophie Georgin-Lavialle

**Affiliations:** ^1^ Sorbonne Université, Service Médecine Interne, Centre de Référence des Maladies Autoinflammatoires et des Amyloses (CEREMAIA), Asssistance Public des Hôpitaux de Paris (APHP), Hôpital Tenon, Paris, France; ^2^ Sorbonne Université, Service de Médecine Interne, Asssistance Public des Hôpitaux de Paris (APHP), Hôpital Saint Antoine, Paris, France

**Keywords:** AOSD, auto-inflammatory disease, myelodysplastic/myeloproliferative disorders, VEXAS syndrome, autoimmunity

## Introduction

With great interest, we read the recent article entitled ‘Case Report: A Rare Case of Elderly-Onset Adult-Onset Still’s Disease in a Patient With Systemic Lupus Erythematosus’, published online in your journal. In this paper, Hirooka et al. reported the case of an 80-year-old Japanese woman with systemic lupus erythematosus who experienced biological and clinical features compatible with adult-onset Still’s disease (AOSD).

## Discussion

We were challenged firstly by the co-occurrence of two rare diseases and secondly by their very late onset. Occurrence of SLE after 75 years of age is a rare condition, this disease being mainly affecting women of reproductive age between the second and fourth decades of life with late onset (>50 years old) in only 5% to 20% (1, 2). In the past, SLE was reported as part of the autoimmune manifestation of myelodysplastic syndrome (3–5). The patient presented ACR EULAR criteria for SLE, but the natural history with an evolution toward an AOSD like presentation makes us wonder if arguments for myelodysplasia were looked for at this stage, particularly clonal haematopoiesis with myeloid NGS.

Beck et al. (6) with their recent description of VEXAS syndrome (vacuoles, E1 enzyme, X-linked, autoinflammatory, somatic) highlighted the existence of autoinflammatory syndrome of late onset associated with a somatic mutation in *UBA1*, a gene encoding ubiquitin-like modifier activating enzyme 1. Its phenotype is still extending but mostly characterized by fever, skin lesions, lung infiltrates, arthralgia, lymph node, and biological inflammatory syndrome with haematological abnormalities including macrocytic anaemia and myelodysplastic syndrome (7). In 2022, VEXAS is considered as a differential diagnosis for AOSD particularly in the elderly (8) and previous diagnosis of AOSD have been rectified a posteriori with *UBA1* screening (9).

In addition to the late onset of AOSD and the existence of autoimmunity, cutaneous signs appeared atypical. The authors rightly noticed the works of Maruyama et al. and Mollaeian et al. (10, 11) about atypical skin rash reported in early-onset AOSD, but these patients did not benefit from screening for VEXAS either. Moreover, the oedema described on both eyelids in the case reported reminds us of the 10 patients with periorbital oedema over the 116 VEXAS syndromes from the French cohort but also the 4 patients identified as undifferentiated systemic autoinflammatory disorder in our recent work (8, 12).

Therefore, we think it would be useful to know the red blood cell mean corpuscular volume to consider macrocytosis or not but also more intel about the bone marrow, especially the presence or absence of vacuoles in myeloid progenitors. Bone marrow showed no finding of malignant disease, but has exploration for clonal haematopoiesis been done with myeloid NGS? If not, it would be a true asset for diagnosis exploration as far as screening for *UBA1* mutation in Sanger technique is concerned.

Finally, we have of course noticed that the patient was a woman, making the probability diagnosis for VEXAS syndrome, an X-linked disease, low but with a few cases reported in women (7, 13) thanks to X monosomy, skewed X inactivation, uniparental disomy or biallelic mutation. Female chromosome X mosaicism is age-related and preferentially affects the inactivated X chromosome. This patient could also have one of the ‘VEXAS-like diseases’, with late-onset autoinflammatory disease, part of the actual USAID, secondary to a clonal haematopoiesis whose mutation we are going to identify in the future.

## Author Contributions

DM wrote the manuscript. All authors contributed to the article and approved the submitted version.

## Conflict of Interest

The authors declare that the research was conducted in the absence of any commercial or financial relationships that could be construed as a potential conflict of interest.

## Publisher’s Note

All claims expressed in this article are solely those of the authors and do not necessarily represent those of their affiliated organizations, or those of the publisher, the editors and the reviewers. Any product that may be evaluated in this article, or claim that may be made by its manufacturer, is not guaranteed or endorsed by the publisher.

## Abbreviations

AOSD, adult-onset Still’s disease; UBA1, ubiquitin-like modifier activating enzyme 1; VEXAS, vacuoles, E1 enzyme, X-linked, autoinflammatory, somatic syndrome.
